# Neural regulation of ILC2s in allergic airway inflammation

**DOI:** 10.3389/falgy.2022.1094259

**Published:** 2023-01-10

**Authors:** Christopher M. Thomas, R. Stokes Peebles

**Affiliations:** ^1^ Division of Allergy, Pulmonary, and Critical Care Medicine, Vanderbilt University Medical Center, Nashville, TN, United States; ^2^ Department of Pathology, Microbiology, and Immunology, Vanderbilt University School of Medicine, Nashville, TN, United States; ^3^ Research Service, Tennessee Valley Healthcare System, United States Department of Veterans Affairs, Nashville, TN, United States

**Keywords:** adrenergic, cholinergic, immunity, neuropeptides, neurotransmitters, ILC2

## Abstract

The sympathetic nervous system (SNS) and parasympathetic nervous system (PNS) regulate the effector functions of group 2 innate lymphoid cells (ILC2s) through *β*2 adrenergic receptor (ADRB2) and nicotinic/muscarinic cholinergic receptor signaling, respectively. To further maintain the critical balance between host-protective and pathogenic type 2 inflammation in the lungs, neuropeptides neuromedin B (NMB) and neuromedin U (NMU) function to suppress or promote ILC2 responses in synergy with IL-33/IL-25, respectively. Additionally, the release of ATP into the extracellular environment in response to cell death caused by challenge to the airway epithelial barrier quickly becomes converted into adenosine, which helps keep the inflammatory response in check by suppressing ILC2 responses. Besides neurotransmitter and neuropeptides derived from other cells, ILC2s further regulate allergic airway inflammation through the production of acetylcholine (ACh) and calcitonin gene-related peptide (CGRP). In this article we review the neuromodulation of ILC2s through cholinergic and adrenergic signaling, neuropeptides, and adenosine and its role in allergic airway inflammation. Furthermore, we discuss the potential clinical utility of targeting these pathways for therapeutic goals and address directions for future research.

## Introduction

The autonomic nervous system consists of two distinct systems: the sympathetic (S-) and parasympathetic (P-) nervous systems (NS) (1). The SNS innervates nearly every tissue and uses norepinephrine (NE) which acts on adrenergic receptors to initiate the “fight-or-flight” response. The PNS uses acetylcholine (ACh) that acts on muscarinic (m-) and nicotinic (n-) cholinergic receptors (AChR) to activate the “rest-and-digest” response (1).

The vagus nerve is the major cholinergic nerve of the PNS that innervates human viscera, including the lungs (2). Upon electrical stimulation, the vagus nerve released ACh which promoted airway remodeling, mucus secretion, airway inflammation, and bronchoconstriction in both mouse and guinea pig models, contributing to the pathogenesis of chronic obstructive pulmonary disease (COPD) and asthma (3). Despite ACh's role in COPD, animal models of sepsis, cystic fibrosis, ulcerative colitis, and arthritis revealed its ability to attenuate the development of disease pathology by acting on macrophages and lymphocytes (4–8). However, long-acting anticholinergics used to treat patients with COPD have shown promise in their therapeutic use for patients with asthma (3). One of the major contributors to the pathology of asthma are type 2 cytokines: interleukin (IL)-4, IL-5, IL-9, and IL-13 released by CD4^+^ type 2 helper T (Th2) cells and group 2 innate lymphoid cells (ILC2s). ILC2s express both n- and mAChR; the *β*2 adrenergic receptor (ADRB2); adenosine receptor A2A; and neuropeptide neuromedin U and B (NMU and NMB) receptors NMUR1 and NMBR, respectively; allowing for neuromodulation of ILC2 effector responses (Table 1). Additionally, ILC2s also express tryptophan hydroxylase 1, choline acetyltransferase (ChAT), and *Calca*—enzymes used for the synthesis of serotonin (9), ACh (10), and calcitonin-gene related peptide (CGRP) (11), respectively.

**Table 1:** Biological function of neurotransmitters and neuropeptides in mice.

Peptide	Neurotransmitter/Neuropeptide	Source	Receptor	Location	Biological Function
NE	Neurotransmitter	Adrenal glands	ADRB2	Intestines and Lungs	Negative regulator of ILC2s
Ach	Neurotransmitter	ChAT and Vagal nerves	M3R	Intestines, Lungs, and MLNs	Suppresses airway neutrophilia
GTS-21/PNU-282987	Neurotransmitter ACh surrogate	Synthetically derived	*α*7nAChR	Lungs	Negative regulator of ILC2s
CGRP	Neuropeptide	PNECs	RAMP1 and CALCRL	Lungs	Promotes IL-5, suppresses IL-13
NMU	Neuropeptide	Cholinergic mucosal neurons and CNS	NMUR1	Intestines and Lungs	Positive regulator of ILC2s
NMB	Neuropeptide	Cholinergic mucosal neurons and CNS	NMBR	Lungs	Negative regulator of ILC2s
Adenosine	Neuropeptide	Breakdown of ATP by CD39 and CD73	A2A	Lungs	Negative regulator of ILC2s

A2A, adenosine receptor 2a; α7nAChR, nicotinic acetylcholine receptor α7; ADRB2, beta-2 adrenergic receptor; ACh, acetylcholine; ChAT, choline acetyltransferase; CALCRL, calcitonin receptor like receptor; CGRP, calcitonin gene-related peptide; CNS, central nervous system; GAT, gonadal adipose tissue; GTS-21, 3-(2,4 dimethoxybenzylidene anabaseine); M3R, muscarinic acetylcholine receptor M3; MLNs, mesenteric lymph nodes; NE, norepinephrine; NMU, neuromedin U; NMB, neuromedin B; NMUR1, neuromedin U receptor 1, NMBR, neuromedin B receptor; PNECs, pulmonary neuroendocrine cells; RAMP1, receptor activity modifying protein 1; RET, rearranged during transfection; ILC2s, group 2 innate lymphoid cells.

Herein we review the neuromodulation of ILC2s as it pertains to allergic airway inflammation and discuss the implications of these findings in clinical practice as well as existing gaps with the mentioned studies and directions for future research.

## Adrenergic neurotransmitter signaling

Human and mouse ILC2s aggregate around adrenergic neurons in the small intestinal lamina propria (siLP) and lungs, and express ADRB2—a receptor for epinephrine and NE. Interestingly, expression of *Adrb2* on siLP ILC2s was reduced after subcutaneous *N. brailiensis* (*Nb*) infection when compared to naïve mice, revealing a dynamic mechanism that responds to inflammatory cues. To determine its function in lung ILC2 biology, *Adrb*2^+/+^ and *Adrb*2^−/−^ mice were generated and intranasally challenged with either IL-33 or *Alternaria alternata* (*AA*) extract. Following challenge, *Adrb*2*^−/−^* mice exhibited greater frequencies of ILC2s compared to *Adrb*2*^+/+^* mice. Importantly, ILC2 frequencies in the lungs became reduced in C57BL/6J (B6) mice given clenbuterol, a ADRB2 agonist, intraperitoneally compared to the vehicle-treated control. Expression of the proliferation marker Ki67 in mice infected with *Nb* was reduced upon clenbuterol treatment compared to the vehicle-treated control, despite comparable frequencies of apoptotic ILC2s. As such, ADRB2 signaling serves as a negative regulator of ILC2s by suppressing their proliferation in the lungs and intestines of mice (12). To further validate this finding, irradiated B6 mice subcutaneously infected with *Nb* and reconstituted with *Adrb*2*^−/−^* and *Adrb*2*^+/+^* bone marrow (BM) had greater frequencies of *Adrb*2*^−/−^* ILC2s compared to *Adrb*2*^+/+^* ILC2s. Importantly, no differences in ILC2 frequencies existed in these chimeric mice prior to infection (12).

## Cholinergic neurotransmitter signaling

ChAT^+^ ILC2s produce ACh as determined by high-performance liquid chromatography and mass spectrometry (13). To determine the location of ChAT^+^ cells, ChAT-eGFP^BAC^ reporter mice that express enhanced green fluorescent protein (eGFP) under the control of ChAT were used (14). Starting on day (D)4 after subcutaneous inoculation of *Nb*, ChAT^+^ CD45^+^ cells expanded in the lungs compared to the naïve control mice. Of those CD45^+^ cells, lung ILC2s expressed the greatest amount of ChAT-eGFP at D4, peaking at D7, and remaining elevated at D21 in both the tissue and bronchoalveolar (BAL) fluid. ChAT-eGFP^BAC^ mice intranasally challenged with *AA* extract developed pulmonary eosinophilia and increases in ChAT-eGFP^+^ CD4^+^ T cells, natural killer (NK) cells, and ILC2s 24 h after challenge, compared to the PBS-challenged controls. ILC2s displayed the greatest elevation of ChAT-eGFP, revealing that type 2 inflammation upregulated the cholinergic phenotype in ILC2s. To determine the role of ChAT during inflammation, *Rora*^Cre+^*ChAT*^LoxP^ mice were generated which knocks out *ChAT* under the control of *Rora* (RAR-related orphan receptor alpha)—a nuclear receptor that is important for ILC2 development. Upon subcutaneous inoculation of *Nb*, *Rora*^Cre+^*ChAT*^LoxP^ mice exhibited reduced IL-5 and IL-13 levels, pulmonary eosinophilia, and airway mucin in total lung tissue at D6 post infection (p.i.) compared to the infected ChAT^LoxP^ control. Furthermore, fewer ILC2s were found in the lungs and MLNs of *Rora*^Cre+^*ChAT*^LoxP^ mice after *Nb* infection. Flow analysis revealed that *Rora*^Cre+^*ChAT*^LoxP^ ILC2s express less Ki67, inducible T-cell co-stimulator (ICOS), and ST2 than their control counterpart, revealing a decreased activation state. Importantly, the effects of ChAT deletion are ILC2 specific as the effector and proliferative capacities of lung CD4^+^ T cells which also produce ACh and express RORɑ (15) remained unaffected between the two genotypes (13). Conversely, *Rora*^Cre+^*ChAT*^LoxP^ mice intranasally challenged with *AA* extract exhibited more inflammatory M1 macrophages and neutrophil chemo-attractants CXCL1/CXCL2, while maintaining similar eosinophil, ILC2, and type 2 cytokine levels in the BAL fluid (16). These findings reveal that ChAT induced ACh production in ILC2s is critical for ILC2-mediated parasite clearance and prevention of airway neutrophilia.

Interestingly, *Nb* produces acetylcholinesterase (AChE)-the enzyme that degrades ACh, a likely mechanism used to evade host immunity (17, 18). BALB/c mice intranasally challenged with *AA* extract and active secretory AChE derived from *Nb* had increased CXCL1/CXCL2 levels but reduced eosinophilia, M2 macrophages, and IL-5 and IL-13 levels in the lung tissue compared to both the PBS- and inactive AChE-treated controls. However, active AChE administration at baseline increased IL-5 and IL-13 levels from lung ILC2s, and M2 macrophage numbers. Overall, these results suggest that ACh suppresses airway neutrophilia by reducing CXCL1/CXCL2 levels and may play an in-direct role in negatively regulating ILC2-mediated allergic airway inflammation (16).

Using end point PCR and gel electrophoresis, expression of both mAChR and nAChR subtypes were detected on ILC2s from the lungs of *Nb*-challenged ChAT-eGFP^BAC^ mice. To determine the receptor(s) through which ACh acts, WT ILC2s isolated from the lungs of *Nb*-infected B6 mice were cultured with IL-7 and IL-2, with or without the selective M3 mAChR (M3R) antagonist 1,1-dimethyl-4-diphenylacetyl piperidinium oxide (4-DAMP) or the non-selective nAChR antagonist mecamylamine. Interestingly, 4-DAMP restricted ILC2 proliferation while mecamylamine had no effect, revealing that ACh likely signals through mAChR expressed on ILC2s in an autocrine fashion to initiate expansion and type 2 immunity (13).

## Cholinergic therapeutic targeting

The PNS is responsible for enhancing asthma exacerbations by promoting bronchoconstriction. Tiotropium bromide is a long-acting M3 mAChR (M3R) antagonist (LAMA) used for the treatment of asthma and COPD. Due to the heterogeneity of asthmatic diseases, LAMA may be combined with inhaled corticosteroids and/or a long-acting ∝2 agonist to prevent the risk of asthmatic exacerbations in humans (3, 19, 20). Additionally, tiotropium suppressed eosinophils; IL-4, IL-5, and IL-13 production; and mucus hyperproduction in mice intranasally challenged for 3 consecutive days with papain compared to vehicle-treated controls, as well as reduced ILC2 and CD4+ T cell numbers in the BAL fluid despite no affect in alarmins. Importantly, tiotropium had no effect on lung ILC2 cytokine production when cultured with IL-33 alone; however, upon the addition of basophils, IL-4, IL-5 and IL-13 levels decreased. Western blot and RNA sequence revealed that basophils highly express M3R and produce IL-4 upon papain challenge, an effect attenuated by tiotropium. Importantly, tiotropium also suppressed Il-4, IL-5, and IL-13 levels in human IL-33 stimulated basophil cultures. Overall, these studies revealed that tiotropium indirectly attenuates ILC2-mediated type 2 inflammation in the lungs of mice and humans by blocking M3R signaling on basophils (21).

In addition to M3R, ACh also signals through the alpha 7 nicotinic ACh receptor (*α*7nAChR). Isolated ST2^+^ ILC2s cultured with IL-33 exhibited a higher expression of *α*7nAChR, but not *α*4nAChR, as determined by quantitative real-time polymerase chain reaction (qRT-PCR) and flow cytometry in mice challenged with recombinant mouse (rm)IL-33 or rmIL-25 intranasally compared to the PBS control. To determine the significance of this, mouse lung ILC2s were isolated and cultured with increasing doses of GTS-21 (*α*4 and *α*7nAChR agonist) in the presence of rmIL-33, rmIL-2, and rmIL-7. Interestingly, IL-5 and IL-13 production was attenuated in a dose dependent manner, suggesting that GTS-21 may suppress type 2 cytokine production. Rag2^−/−^ *Il*2*rg*^−/−^ mice, which lack B, T, and NK cells, reconstituted with WT ILC2s and intranasally challenged with IL-33 and GTS-21 and exhibited reduced airway hyperreactivity (AHR) and eosinophilia compared to the mice reconstituted with *α*7nAChR-deficient ILC2s. Gene analysis on GTS-21 treated ILC2s revealed reduced IKK*α*/*β* phosphorylation and subsequent reduced expression of NF-*κ*B, a critical transcriptional regulator required for GATA-3 expression and ILC2 survival. Additionally, Rag2^−/−^ *Il*2*rg*^−/−^ mice that were reconstituted with human ILC2s and challenged with recombinant human (rh)IL-33 intranasally had reduced AHR and allergic airway inflammation upon GTS-21 treatment after 3 consecutive days. Overall, the results in mice show a therapeutic potential for GTS-21 to suppress ILC2-mediated allergic responses in humans (22). However, because GTS-21 binds ɑ4nAChR with a higher affinity than *α*7nAChR, unwanted side effects such as heightened anxiety, D3 dopamine receptor downregulation, and increased growth hormone release could occur (23–25). As a result, PNU-282987 was investigated due to its higher affinity to *α*7nAChR. Interestingly, mice intranasally challenged with *AA* extract or IL-33 and treated with GTS-21 or PNU-282987 both had equivalently attenuated goblet cell hyperplasia, eosinophil and ILC2 migration, and type 2 effector cytokines. Additionally, ILC2s isolated from mouse lungs cultured with IL-2 and IL-7 with PNU-282987 or GTS-21 had equal reduction of Ki67 and GATA-3 despite IL-33 presence. However, though both PNU-282987 and GTS-21 inhibited IKK phosphorylation and NF-*κ*B p65 after 24hr treatment, the inhibitory effect of PNU-282987 was much greater suggesting that PNU-282987 might serve as a better asthmatic therapeutic compared to GTS-21 due to its higher binding affinity to *α*7nAChR and greater inhibition of IKK phosphorylation (26).

## Neuropeptides

NMU is a neuropeptide produced by mucosal cholinergic neurons that signals through NMU receptor 1 (NMUR1) which is highly expressed on ILC2s in both mice and humans as determined using *Nmur*1*^iCre−eGFP^* reporter mice and genome-wide transcriptional analysis, respectively (27–29). Interestingly, *Nmur*1^−/−^ mice intranasally challenged with house dust mite (HDM) had reduced ILC2 frequencies compared to the PBS-challenged control. B6 mice only showed a significant upregulation in AHR, eosinophilia, ILC2s, and type 2 cytokine production in the lung and BAL fluid when intranasally challenged with NMU and IL-33 or IL-25 together than when alone. Flow cytometry and qRT-PCR revealed that NMU amplified the expression of *Il17rb* on ILC2s, which encodes the receptor for IL-25, thus revealing that IL-33/IL-25 + NMU promotes allergic airway inflammation in mice (30).

In culture, NMU also upregulated the expression of *Ramp*1 and *Calcr*1 in ILC2s, which encode the co-receptors that make up the calcitonin-gene related peptide (CGRP) receptor, when co-cultured with IL-33 (11). Pulmonary neuroendocrine cells (PNECs) are rare airway epithelial cells that produce CGRP and are the only source of *γ*aminobutyric acid (GABA) in the lungs of mice (31, 32). PNECs reside within airway branch junctions where it is speculated that they function to sample inhaled particulate matter and express *Ascl*1 (also known as *Mash*1), a gene that encodes the basic helix-loop-helix transcriptional factor Mash1 which is essential for their differentiation (33). To determine the role of PNECs in immunity, cre was inserted into the *sonic hedgehog gene* (*Shh^cre^*) to inactivate *Ascl1* only in PNEC precursors to bypass perinatal lethality, thus creating *Ascl1*CKO mice. At baseline, *Ascl1*CKO mice did not exhibit defects in lung cell development, but did exhibit reduced CGRP and GABA levels, goblet-cell hyperplasia, immune-cell infiltration, ILC2s, eosinophils, and Th2 cells either after intranasal house dust mite (HDM) challenge or intraperitoneal sensitization followed by aerosolized challenge of ovalbumin (OVA), despite having similar alarmin levels in the lungs. Immunofluorescent CGRP staining of mouse lung epithelium revealed restricted expression in PNECs after OVA challenge and localization of ILC2s near PNECs in regions called nodal points, where they can respond to CGRP and GABA. Despite this, GABA had no effect on IL-5 production while CGRP elevated IL-5 levels in naïve mouse lung ILC2 cultures, but only in the presence of IL-33 or IL-25. Disruption of GABA signaling through the generation of *Gad1* (encodes GABA synthesis protein) or *Vgat* (encodes GABA transporter) knockout mice caused deficient goblet cell hyperplasia upon OVA sensitization and challenge, supporting a role of GABA in goblet cell hyperplasia. Additionally, OVA-challenged *Ascl1*CKO mice had restoration in asthma-like phenotypes such as goblet cell hyperplasia, immune cell infiltration, and type 2 cytokine production upon intratracheal administration of CGRP and GABA. Lung sections from asthmatic patients stained with anti-CGRP revealed increased CGRP^+^ PNECs compared to healthy controls. Together, these findings suggest that PNECs promote asthmatic responses in humans and mice through the production of CGRP and GABA (34). Additionally, *Il5*^hi^ ILC2s also produced CGRP and expanded upon IL-33 intranasal challenge in mice. Interestingly, Rag2^−/−^ mice challenged with IL-33 and CGRP had reduced immune-cell infiltrates, eosinophilia, goblet cell hyperplasia, and type 2 cytokine levels in the lung tissue and BAL fluid compared to the IL-33 treated control. Furthermore, *Ramp1^−/−^* mice intranasally challenged with IL-33 had increased type 2 cytokine levels, ILC2 levels, and eosinophils after intranasal IL-33 treatment which failed to be resolved by CGRP treatment. Collectively, these results support a negative regulatory role of CGRP (11, 35). As a result, it appears that CGRP has a complex role in regulating type 2 allergic airway inflammation that may be dependent on its source (ILC2s vs. PNECs) and or environmental cues (IL-33 vs. GABA).

In addition to ILC2s, basophils also serve as important mediators of allergic airway inflammation. Interestingly, ILC2s isolated from basophil-depleted mice had reduced NMBR expression 7 days after subcutaneous inoculation of *Nb*, as determined using RNA-seq. Recombinant (r)NMB administration to Rag2^−/−^ mice infected with *Nb* reduced ILC2 responses, mucus production, and eosinophilia which lead to increased alveoli pathology and failure to clear worms compared to the vehicle-treated mice. ILC2s cocultured with basophils had elevated *Nmbr* expression and type 2 cytokine production. To determine how basophils upregulated *Nmbr* expression and type 2 cytokine production, basophil effector molecules IL-4 and PGE_2_ were added to ILC2 cultures. In doing so, IL-4 had no effect while PGE_2_ elevated NMBR expression. Upon the addition of PGE_2_ and NMB to ILC2 cultures, IL-5 and IL-13 became attenuated while NMBR expression was increased. Overall, these results suggest that PGE_2_ from basophils primed NMU-induced suppression of ILC2s by upregulating NMBR surface expression (36).

## Adenosine signaling

Adenosine results from the breakdown of exogenous ATP released from cells upon death and accumulates in the central nervous system where it promoted sleep and prevented cerebral ischemia, epilepsy, Alzheimer's, Parkinson's, and various sleep disorders by binding to its receptors apart of the G-protein coupled receptor family (37). One of Adenosine's receptors, A2A, became highly expressed on lung ILC2s in mice intranasally challenged with papain or IL-33 compared to the naïve control. To determine A2A's role in ILC2 function, lung ILC2s from A2A^−/−^ mice were adoptively transferred into triple immunodeficient NCG (NOD CRISPR *Prkdc Il*2*r* gamma) mice, which lack functional/mature adaptive immune cells and have reduced macrophages and dendritic cells. After 3 consecutive days of intranasal IL-33 challenge, NCG mice reconstituted with A2A^−/−^ ILC2s had elevated ILC2s, eosinophils, and type 2 cytokines in the BAL fluid compared to the control mice reconstituted with WT ILC2s. Furthermore, Rag1^−/−^ mice which lack mature B and T cells, intranasally challenged with papain for 5 consecutive days and treated with NECA (adenosine analog) exhibited reduced eosinophil infiltrate, ILC2s, and type 2 cytokines in the BAL fluid compared to the vehicle-treated control. As a result, NECA/adenosine targeted A2A on ILC2s to suppress ILC2-mediated type 2 inflammation which was achieved by cAMP-induced inhibition of NF-*κ*B as determined by gene analysis (38).

## Discussion

Here we reviewed the clinical utility of ACh and the implications of neuronal signaling on ILC2s as it pertains to allergic airway inflammation. We noticed a duality in the role ACh plays in regulating ILC2s that seemed to be dependent on its target receptor (M3R vs. *α*7nAChR). To prevent redundancy of ACh signaling, ILC2s likely express M3R and *α*7nAChR at different levels which depend on environmental factors. CGRP also exhibits duality, suppressing IL-33 + NMU-ILC2 activation but restoring asthma-like phenotypes in OVA-challenged PNEC-deficient mice given GABA. Additionally, ChAT^+^ ILC2s were crucial for effective parasitic clearance but not allergic airway inflammation. Collectively, these results reveal the potential environmental influence on neuronal ILC2 regulation and thus should be further investigated in future studies.

Many of the experiments argue changes in allergic airway inflammation resulting at the ILC2 level. However, these experiments used global knockout models (e.g., *Adrb*2^−/−^, *Nmur*1*^−/−^*, and *Ramp*1*^−/−^*) and pharmacological inhibitors and agonists as methods to support their arguments. As such, it is difficult to discern whether the clinical phenotypes observed resulted from direct changes to ILC2s or indirect changes arising from other tissues and or immune cells affected. Future experiments need to combat this issue by utilizing conditional knockout mouse models that specifically delete genes of interests in only ILC2 populations. In the past this technology wasn't available, but *Nmur*1*^iCre−eGFP^Rosa*26*^LSL−RFP^* mice revealed that *Nmur*1 is selectively expressed in ILC2s and could serve as a specific marker used to generate ILC2 knockout models. With this idea in mind, *Nmur*1*^iCre−eGFP^* mice were crossed with *Id*2*^fl/fl^* and *Gata*3*^fl/fl^* mice—both of which encode essential transcription factors important for the development and maintenance of ILC2s. In doing so, *Nmur*1*^iCre−eGFP^Id*2*^fl/fl^* and *Nmur*1*^iCre−eGFP^Gata*3*^fl/fl^* mice were generated and observed to both lack ILC2s while having no noticeable effect on other immune cell types besides eosinophils, the latter resulting from deficient eosinophil maturation, proliferation, and survival. As a result, these mice may prove to be a good conditional knockout model that will help advance our knowledge on ILC2s and their role in allergic airway inflammation (39).

## Conclusion

**Figure 1:**
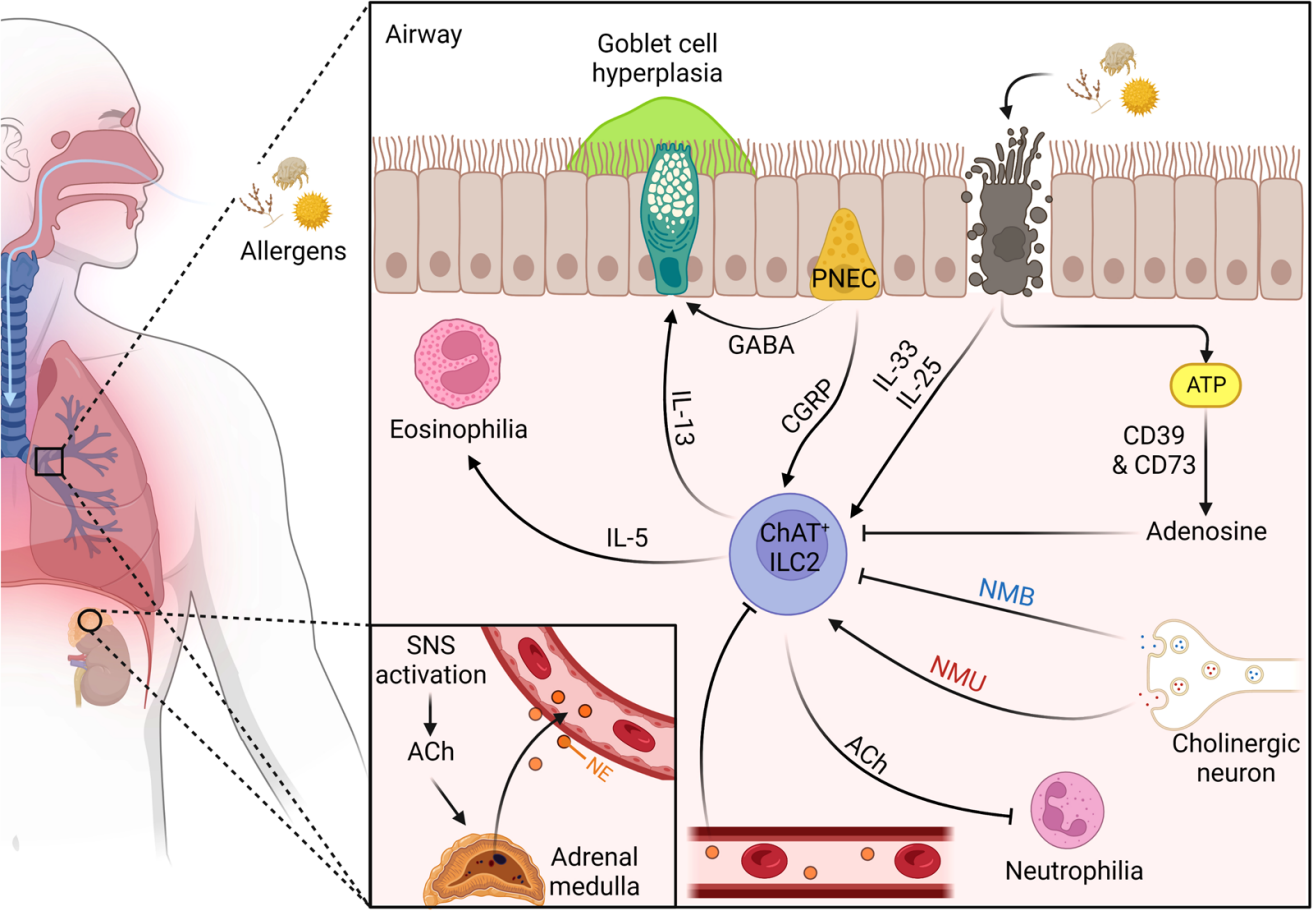
The dynamic neuro-regulation of ILC2s in the lungs upon allergen challenge. (1) Inhaled allergen enters through the nasal cavity and makes its way to the distal airway. (2) PNECs located at branch junctions detect the allergen and release CGRP and GABA, promoting IL-5 release from ILC2s and goblet cell hyperplasia, respectively. (3) The proteolytic allergen disrupts tight junctions between epithelial cells leading to their death and the release of alarmins IL-25 and IL-33, and exogenous ATP. (4) exogenous ATP is quickly degraded into adenosine by ectonucleotidases CD39 and CD73 to suppress ILC2s. (6) IL-25/IL-33 activates the conical type 2 cytokine pathway promoting eosinophilia and goblet cell hyperplasia, as well as upregulates ChAT expression. (7) ChAT + ILC2s secrete ACh which suppresses airway neutrophilia. (8) Mucosal cholinergic neurons release NMU and NMB in response to allergen challenge. (9) NMU and IL-25 work synergistically to amplify type 2 inflammation. (10) NMB controls allergic type 2 inflammation by suppressing IL-5 and IL-13 release. (11) Allergen challenge leads to sympathetic activation and the release of ACh which promotes the adrenal medulla to secrete NE into the bloodstream where it travels to the lungs to suppress ILC2s. Figure 1 was created using BioRender (https://biorender.com/). Abbreviations: ACh, acetylcholine; CGRP, calcitonin gene-related peptide; ChAT, choline acetyltransferase; GABA, *γ*-aminobutyric acid; IL, interleukin; ILC2, type 2 innate lymphoid cell; NE, norepinephrine; NMB, neuromedin B; NMU, neuromedin U; PNEC, pulmonary neuroendocrine cell; SNS, sympathetic nervous system.

Neuronal signals serve as an efficient method used to maintain a balance between ILC2-mediated host protective and pathogenic type 2 inflammation in the lungs of humans (Figure 1) and mice. As such, this field warrants further investigation in its potential to generate better immune targeted therapies at the neural level.
